# Approaching a diagnostic point-of-care test for pediatric tuberculosis through evaluation of immune biomarkers across the clinical disease spectrum

**DOI:** 10.1038/srep18520

**Published:** 2016-01-04

**Authors:** Synne Jenum, S. Dhanasekaran, Rakesh Lodha, Aparna Mukherjee, Deepak Kumar Saini, Sarman Singh, Varinder Singh, Guruprasad Medigeshi, Marielle C. Haks, Tom H. M. Ottenhoff, Timothy Mark Doherty, Sushil K. Kabra, Christian Ritz, Harleen M. S. Grewal

**Affiliations:** 1 Department of Global Public Health and Primary Care, University of Bergen, and Department of Medical Microbiology, Vestre Viken Hospital Trust, Drammen, Norway; 2 Department of Clinical Science, Faculty of Medicine and Dentistry, University of Bergen, N-5021, Norway; 3 Department of Microbiology, Haukeland university hospital, University of Bergen,N-5021, Norway; 4 Department of Pediatrics, All India Institute of Medical Sciences, New Delhi, India; 5 Division of Clinical Microbiology & Molecular Medicine, Department of Laboratory Medicine, All India Institute of Medical Sciences, New Delhi, India; 6 Department of Pediatrics, Kalawati Saran Children Hospital, New Delhi, India; 7 Department of Infectious Diseases Group, Immunology and Immunogenetics of Bacterial Infectious Disease, Leiden University Medical Center, The Netherland; 8 GlaxoSmithKline Pharma, Vaccines, Brøndby, Denmark; 9 Department of Nutrition, Exercise and Sports, University of Copenhagen, Denmark

## Abstract

The World Health Organization (WHO) calls for an accurate, rapid, and simple point-of-care (POC) test for the diagnosis of pediatric tuberculosis (TB) in order to make progress “Towards Zero Deaths”. Whereas the sensitivity of a POC test based on detection of *Mycobacterium tuberculosis* (MTB) is likely to have poor sensitivity (70–80% of children have culture-negative disease), host biomarkers reflecting the on-going pathological processes across the spectrum of MTB infection and disease may hold greater promise for this purpose. We analyzed transcriptional immune biomarkers direct *ex-vivo* and translational biomarkers in MTB-antigen stimulated whole blood in 88 Indian children with intra-thoracic TB aged 6 months to 15 years, and 39 asymptomatic siblings. We identified 12 biomarkers consistently associated with either clinical groups “upstream” towards culture-positive TB on the TB disease spectrum (*CD14, FCGR1A, FPR1, MMP9, RAB24, SEC14L1*, and *TIMP2)* or “downstream” towards a decreased likelihood of TB disease (*BLR1, CD3E, CD8A, IL7R*, and *TGFBR2*), suggesting a correlation with MTB-related pathology and high relevance to a future POC test for pediatric TB. A biomarker signature consisting of *BPI, CD3E, CD14, FPR1, IL4, TGFBR2, TIMP2* and *TNFRSF1B* separated children with TB from asymptomatic siblings (AUC of 88%).

Tuberculosis (TB) remains one of the world’s major public health problems. Following the declaration of TB as a global emergency in 1993, the response focused almost exclusively on reducing the transmission of *Mycobacterium tuberculosis* (MTB)^1^. Case finding and treatment in the pediatric population were not prioritized within National TB Programs (NTPs), as children were perceived as less contagious. WHO recently launched a strategy for combating pediatric TB, in which an urgent need for research aiming to develop an effective diagnostic test for TB in children, is expressed^2^. Underdiagnosis is currently the rule in pediatric TB, although overdiagnosis also occurs^3^. The consequences are evident both as increased TB related morbidity and mortality or as unnecessary burdens of long-term treatment imposed on families and health care systems. This can be avoided with an accurate diagnostic test that should provide a rapid, sensitive, and specific result to enable early initiation of treatment at the point-of-care (POC); “a diagnostic POC test”. A clear advantage, particularly in resource-limited settings, is the likely reduction in cases lost to treatment by eliminating the need for a repeated visit^4^.

A microbiologically confirmed diagnosis of pediatric TB is indeed challenging due to the paucibacillary nature of disease which results in 70–80% of clinical cases remaining culture-negative even under optimal study conditions. This implies that despite successful implementation of the GeneXpert MTB/RIF assay in 22 high-burden countries^5^, even direct identification of MTB in specimens by nucleic acid amplification tests, like the GeneXpert MTB/RIF assay, will never meet the requirements of a POC test for pediatric TB^6^. Currently, light or fluorescent microscopy of acid-fast stained specimens (sputum/gastric lavage) is still the only diagnostic method available in many primary diagnostic centers when TB is suspected^7^. However, the sensitivity of microscopy is far from satisfying; as low as 34% of culture-confirmed cases in some studies^8^. Therefore, it is likely that POC tests based on host immune responses will be more sensitive in detecting paucibacillary/culture-negative disease in children.

On the road towards a POC test, host immune biomarkers should ideally be explored on platforms that can be easily translated to primary diagnostic centers. Therefore, studies, which build upon already established tools like the IFN-γ release assays, with MTB-specific *ex-vivo* stimulation of whole blood (WB), have been considered extensively, but studies in children are still limited. (Reviewed by Chegou *et al.*^9^). Another approach is genome-wide analysis of RNA expression in host blood direct *ex-vivo* (unstimulated)^10^,^11^,^12^, and recently two studies in children have been published based on three African cohorts^13^ and Warao Amerindians^14^. Genome-wide analysis of transcriptomes are costly and resource-intensive and translation into diagnostic tools in resource-poor settings is challenging, but can be seen as a necessary first step in identifying markers with potential for subsequent refinement as POC tests. To facilitate the search for transcriptional signatures with diagnostic relevance in TB, dual-colour Reverse Transcriptase-Multiplex Ligation-dependent Probe Amplification (dcRT-MLPA) has recently been developed^15^. This method can rapidly profile multiple host genes with a dynamic range and sensitivity comparable to real-time qPCR and RNA sequencing at a cost of 5–7 euros per sample^16^. We have previously explored the diagnostic potential of this assay in Indian children <3 years referred to a TB verification ward for suspected tuberculosis^17^. The limitation of cohort studies of this kind is the small number of sputum-positive TB cases that can actually be expected, even from large cohorts. It is mandatory to include culture-negative pediatric TB cases even though standardized clinical case definition is challenging^18^. In this study representing a population enriched for MTB infection and disease, we therefore used the same methodological approach to identify immune biomarker(s) with a potential to discriminate 1) across the spectrum of MTB infection and disease, and 2) between children with TB and exposed but asymptomatic household siblings. The study samples were selected from a randomized controlled trial of children diagnosed with intra-thoracic TB in Delhi, India. Unstimulated WB was analyzed for transcriptional immune biomarkers direct *ex-vivo* applying dcRT-MLPA, whereas WB stimulated with MTB antigens were analyzed for translational immune biomarkers using an 18-plex multiplex bead array (bioplex).

## Results

### Characteristics of TB cases and asymptomatic siblings

**Figure 1:**
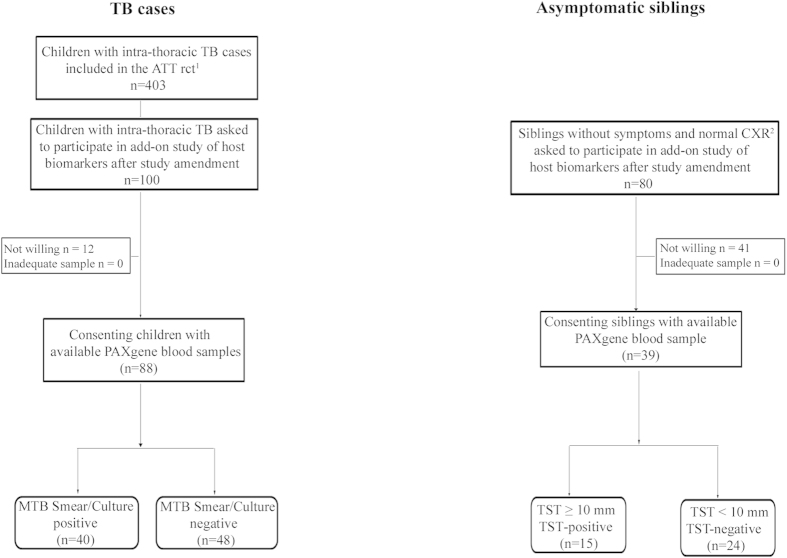
Study flowchart. ¹(A) randomized-controlled trial (rct) of the effect of different micronutrient (MN) supplementation on top of anti-tuberculosis therapy (ATT). The children were assigned to 4 intervention groups; micronutrient supplementation (MN) with or without zinc (Zn), Zn alone or placebo, and followed for 6 months^30^. ^2^Chest X-ray.

The selection and classification of the study subjects are depicted in Fig 1. Characteristics of 88 TB cases and 39 asymptomatic siblings are shown in Table 1 and Table 2, respectively. Age, gender, BCG vaccination status, and exposure to a TB index case (different from the diseased child) were equally distributed between TB cases (culture-confirmed; culture-negative) and asymptomatic siblings (TST-positive; TST-negative). Unfortunately, we do not have BCG data on the siblings, but as parents were likely to provide the same health care for all their children, we judged the BCG status of the diseased siblings to be a good proxy. Among TB cases, cough ≥2 weeks and weight loss was more prominent in culture-confirmed cases (p < 0.001; p = 0.002), supporting more advanced disease in culture-confirmed cases. Interestingly, no TST-positive siblings had known exposure to an adult TB case whereas 7 (28%) of TST-negative children had ≥12 weeks of exposure, 6 with ≥12 hours per day. Furthermore TST-positive siblings were more likely to be underweight (BMI-for-age <5^th^ percentile) than TST-negative siblings, which might suggest increased susceptibility to infection and/or subclinical TB. These findings supported the assumption that TST-negative siblings represented the lower end of the TB disease spectrum, namely innate clearance of MTB infection.

### Comparison of biomarker profiles between the most polar differences in the TB disease spectrum: culture-confirmed TB versus asymptomatic TST-negative household siblings

We started out comparing the groups at the outer ends of the continuous TB disease spectrum: children with intra-thoracic TB established on the basis of culture-confirmed TB, representing the higher end, and asymptomatic TST-negative household siblings representing the lower end of the spectrum. (See Study populations and case definitions in the Methods section).

**Figure 2:**
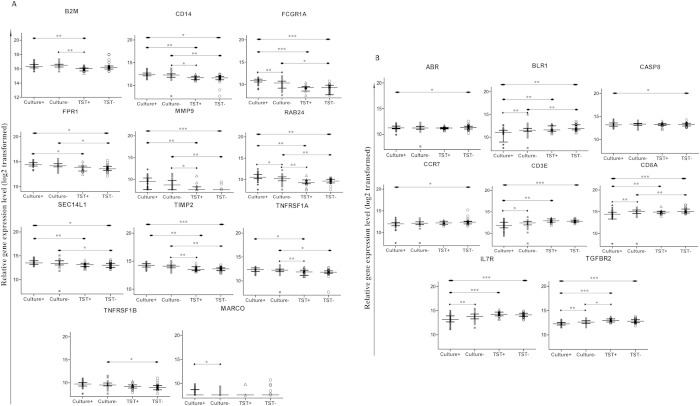
Dot-plot graph depicting genes that are differentially expressed between the four clinical groups: TB cases either culture confirmed (culture + ) or culture negative (culture–), and asymptomatic household siblings either TST-postive (TST + ) or TST-negative (TST–). (**A,B**) illustrates the median with inter quartile range relative gene expression (log 2 transformed) of genes in peripheral blood direct *ex-vivo*. p-value ≤ 0.05 (*), < 0.01 (**), < 0.001 (***) were considered to be significant.

**Figure 3:**
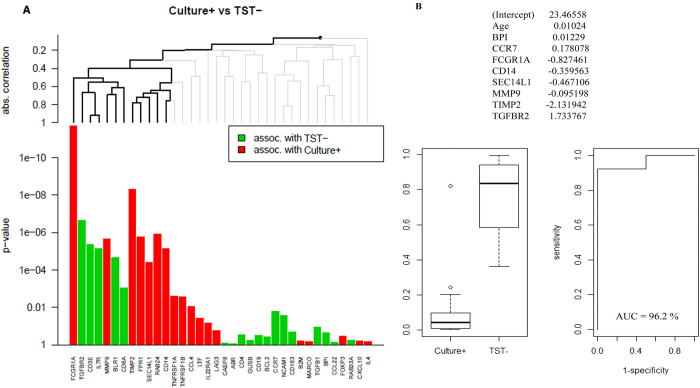
Comparison of dcRT-MLPA gene expression data between culture-confirmed TB cases (culture + ) and asymptomatic TST-negative siblings (TST–) by (**A**) the global test and (**B**) Lasso analysis: The ability of biomarker signatures to predict clinical outcomes were identified following adjustment for age (months). The predicted probability of the identified biomarker signatures to discriminate between the groups is shown by: receiver operator characteristic curves (ROCs), area under the curve (AUC), and box-and-whisker plots (5–95 percentiles).

We first assessed differences in single transcriptional biomarkers (direct *ex-vivo*) (Table 2) and found 7 genes were up-regulated in children with culture-confirmed TB (*CD14, FCGR1A, FPR1, MMP9, RAB24, SEC14L1* and *TIMP2*) compared to TST-negative household siblings (Fig. 2A), and 8 were down-regulated (*ABR, BLR1, CASP8, CCR7, CD3E, CD8A, IL7R*, and *TGFBR2*, Fig. 2B). The complexity of the TB pathogenesis suggests that a POC test must be based on multiple biomarkers^4^, implying a need for biomarker signatures: a global test^19^ confirmed the simultaneous association with culture-confirmed TB for all the 7 up-regulated genes identified by single biomarker analyses. Similarly, 5 of 8 genes were found to be down-regulated in culture-confirmed TB associated with TST-negative household siblings (*BLR1, CD3E, CD8A, IL7R* and *TGFBR2*, Fig. 3A, Table 2). The biomarker signature with the highest potential to discriminate between culture-confirmed TB cases and TST**-**negative household siblings (identified by Lasso regression, AUC 96.2%, Fig. 3B, Table 2), provided further confirmation for the discriminatory role for *CCR7, CD14, FCGR1A, MMP9, SEC14L1, TGFBR2* and *TIMP2*, all identified both by single biomarker analyses and global tests.

There were no significant single nor global differences observed in translational biomarkers between the clinical groups across the TB disease spectrum.

### Comparison of biomarker profiles between clinical groups with less polarity within the TB disease spectrum

Unfortunately, real-life is less clear cut than the categories described above as a large proportion of pediatric TB cases are culture-negative^6^. Keeping in mind the continuous TB disease spectrum^20^, transition to TB in MTB-infected subjects is a concern, particularly in recently exposed young children. Therefore, we aimed to get a broader understanding of the expression of immune biomarkers in children representing different stages of the TB disease spectrum: culture-confirmed TB vs asymptomatic TST-positive household siblings; culture-negative TB vs TST-positive household siblings; culture-negative TB vs TST-negative household siblings and culture-confirmed vs culture-negative TB cases (Table 3; Supplementary Table 2, Supplementary Figure 1 and 2). Comparisons of single biomarkers are shown in Fig. 2. Summarized findings of all conducted analyses including biosignatures as presented in Table 3 (and Supplementary Table 2), clearly illustrating that the largest difference in biomarker profiles were observed between the most polar clinical groups whereas the least differences were observed between neighboring groups, together supporting an “upstream” or “downstream” regulation in line with the TB disease spectrum.

### Differences in biomarker profiles between children with TB disease and TB exposed household siblings: the most relevant approach to a clinical pediatric setting

**Figure 4:**
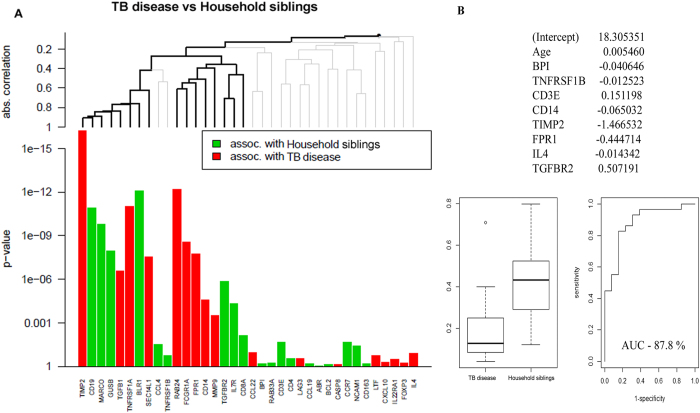
Comparison of dcRT-MLPA gene expression data between TB cases (regardless of culture result) and asymptomatic household siblings (regardless of TST result). by A) the global test and B) Lasso analysis: The ability of biomarker signatures to predict clinical outcomes were identified following adjustment for age (months). The predicted probability of the identified biomarker signatures to discriminate between the groups is shown by: receiver operator characteristic curves (ROCs), area under the curve (AUC), and box-and-whisker plots (5–95 percentiles).

In TB-endemic regions, TB is primarly diagnosed based on symptoms and known TB exposure. A POC test with good discriminatory power between TB disease and other medical conditions is crucial. A large proportion of children seeking healthcare are MTB-infected but ill for other reasons. Therefore, discrimination between TB disease and MTB infection is also an important requirement for a POC test. Accordingly, we assessed differences in biomarker profiles between children with TB disease (regardless of culture result) and asymptomatic household siblings (regardless of TST result) using the same statistical approach. Single biomarker analysis identified 16 differentially expressed biomarkers, of which 12 genes were also identified by a global test (Table 4, Fig. 4A and Supplementary Figure 3A and B). A predictive biomarker signature comprising of 8 biomarkers provided good discriminatory power (Lasso regression, AUC 87.8%; Fig. 4B). Of these 8, 4 genes (*CD14, FPR1, TGFBR2, TIMP2*) were also identified both by single gene analysis and a global test.

## Discussion

In the present study we report a clear pattern of transcriptional biomarkers, analyzed direct *ex-vivo*, across the pediatric TB disease spectrum. As expected, the largest difference in biomarker signatures were observed between the most polar clinical groups: culture-confirmed TB cases versus asymptomatic siblings who remained TST-negative despite prolonged exposure. The smallest differences were observed between neighboring groups on the TB disease spectrum scale. Biomarkers which were consistently associated with clinical groups “upstream” towards culture-positive TB, were *FCGR1A, FPR1, MMP9, RAB24, TNFRSF1A* and *TIMP2*, whereas *BLR1, CD8A, IL7R*, and *TGFBR2* were associated with clinical groups “downstream” towards a decreased likelihood of TB disease. Our study cannot establish whether these biomarkers reflect the cause or consequence of MTB-related pathology, but the clear evidence of correlation with MTB-related pathology considerably strengthen the likelihood of relevance for these biomarkers to a future POC test in children.

The existing evidence for the role of all the analyzed dc-RTMLPA biomarkers in MTB-related pathology is given in Supplementary Table 1.

In this study we describe biomarker signatures with excellent discriminatory power for culture-confirmed TB when compared to asymptomatic TST-negative or TST-positive household siblings (AUC 96% and 95%, respectively). *BPI, CD14, FCGR1A, SEC14L1* and *TGFBR2* were represented in both comparisons. Notably, these biomarker signatures provided superior sensitivity for culture-confirmed TB compared to the Xpert-MTB/RIF performed on gastric lavage^6^. But even more important, when aiming at combating pediatric TB, was the finding of a biomarker signature with a clear diagnostic potential regardless of culture result (*BPI, CD3E, CD14, FPR1, IL4, TGFBR2, TIMP2* and *TNFRSF1B*, AUC of 88%).

The dcRT-MLPA method has been applied in different studies assessing TB biomarkers for different purposes^15^,^16^,^21^,^22^,^23^,^24^,^25^, but few studies have been conducted in children^17^. A recent publication, which provides a proof-of-concept for the use of this assay in a large cohort of adults across four different African populations, identified increased *FCGR1A* expression as the single most consitent classifier of TB disease compared to latent TB infection (LTBI) irrespective of HIV-infection^22^. The clinical groups in the present study which correspond to adults with TB disease (culture-confirmed) and LTBI compared in this large African study, are the children with culture-confirmed TB and the TST-positive asymptomatic siblings. Of the markers identified here (Table 2) *BLR1, FCGR1A, IL7R, SEC14L1, TGFB1* and *TGFBR2*, were all identified in at least two of the African populations and modulated in the same direction. Similarly, a smaller study restricted to Ethiopian adult TB patients and household contacts also identified *BLR1, FCGR1A, IL7R* and *MMP9*^23^ as differentiating markers in line with our findings. Lastly, *BLR1, FCGR1A* and *IL7R* were also differentially expressed between adult TB cases and healthcare workers in Paraguay^16^. This agreement across different populations strengthens the likelihood of these markers as relevant to a future POC test for TB in children. We have previously published the only study in children based on dcRT-MLPA data in another cohort of Indian children <3 years referred for suspected tuberculosis. Of genes differentially expressed between the groups in the present study, only *SEC14L1* and *TGFB1* were also identified as part of the biosignature discriminating between children with clinical TB disease, MTB infection, and uninfected controls^17^. If – as the present study suggests – culture-confirmed TB represents one extreme of the TB disease spectrum, the small number of culture-confirmed patients in that study would reduce the discriminatory power of the analysis, underlining the importance of testing biosignatures in multiple and diverse populations.

A high proportion of the children were underweight according to the BMI-for-age percentile. Malnutrition is both a cause and a consequence of TB disease^26^. We have previously shown that nutritional status affects other immunological read-outs of MTB infection^27^. Therefore, it brings hope for a future POC test based on immunological biomarkers that we detected convincing differences in biomarker profiles despite the lack of correction for nutritional status.

To our knowledge, two previous studies have assessed direct *ex-vivo* host transcriptional signatures in WB in children^13^,^28^. Except for *CD8A*, which was part of the 116 gene set identified in the Warao Amerindian children, none of the identified markers in these studies were included in our dcRT-MLPA panel. However, this does not exclude markers differentially expressed in the present study as relevant to a future POC test. In this regard, Verhagen *et al.*^29^ discovered that when applying their selected biosignature to three other cohorts of adult TB patients that had reported WB gene expression, that their signature first identified in children also discriminated TB from LTBI in the adult cohorts^11^,^12^,^30^. However, while the 116 gene set identified in children retained reasonable discriminatory power in the adult cohorts, the signatures identified primarly in the adult cohorts did not discriminate in the children. Furthermore, all the data sets had their optimal selection of gene sets, which did not overlap, suggesting variation, perhaps at the genetic level and/or due to regional differences in (for example) endemic disease particularly between African and other populations (European, Native American/Colombian)^31^. These findings indicate that biomarker discovery needs to be performed in different populations. Most studies are currently conducted in African countries, because of the high TB incidence rates, but India has the largest number of incident cases in the world (2.0–2.3 million) and should not be forgotten^28^.

The strengths of our study is the significant number of TB cases; both culture-confirmed and culture-negative cases and the fact that household siblings share background factors with the TB diseased children assessed. Based upon clinical characteristics and findings (Table 1 and 2) the selected groups seem adequate to assess the TB disease spectrum. These groups also make our results applicable to diagnostic settings where children are brought to a healthcare facility either because of symptoms or concern that recent TB exposure might cause disease. The possibility of introducing a random sibling effect was corrected for in the analyses of single biomarkers. Notably, children with other inflammatory or infectious conditions may have immune-biomarker profiles more similar to TB cases than household-exposed siblings as direct *ex-vivo* assessment did not include specific antigen stimulation. This aspect needs exploration in future studies.

The relatively small number of household siblings is a limitation of this study, constraining our ability to conclude on biomarker differences at the lower end of the TB disease spectrum (TST-positives versus TST-negatives). However, we have previously reported on this population in another, larger cohort of Indian children, confirming “upstream” expression of *FPR1, TNFRSF1A*, and *TIMP2* in MTB-infected^29^. We acknowledge that lack of adjustments for multiplicity when using statistical test procedures increased the risk of false positive findings, but this liberal approach is warranted because of the exploratory nature of the study. The fact that many of the identified markers correspond to findings in previous studies in adults support the hypothesis that we have detected true differences related to disease extent between the groups. Moreover, the use of global tests and lasso regression, both of which considered biomarkers simultaneously, reduced the need for multiplicity corrections.

In conclusion, the present study identified 12 biomarkers that were consistently associated with either clinical groups “upstream” towards culture-positive TB on the TB disease spectrum (*CD14, FCGR1A, FPR1, MMP9, RAB24, SEC14L1*, and *TIMP2)* or “downstream” towards a decreased likelihood of TB disease (*BLR1, CD3E, CD8A, IL7R*, and *TGFBR2*). This evidence of consistent correlation with MTB-related pathology indicated relevance of these biomarkers in a future POC test for pediatric TB. Furthermore, we report on specific biomarker signatures that could fulfill the requirements for a POC test, which, if validated, would contribute significantly towards a reduction of the annual worldwide TB attributable deaths in children.

## Materials and Methods

### Source population

This study is cross-sectional and partly nested within a randomized, double-blind prospective controlled trial conducted from January 2008 to June 2012 at the All India Institute of Medical Sciences and Kalawati Saran Children Hospital associated with Lady Hardinge Medical College in Delhi, India, for which the study details are described elsewhere^30^. Briefly, children aged 6 months to 15 years who presented with any of: cough and/or fever ≥2 weeks with no improvement after a 7–10 day course of amoxicillin; recent unexplained weight loss/failure to thrive; fatigue/lethargy (reduced playfulness), or; subtle clinical symptoms, and a history of close contact with an adult patient with TB, were screened for TB at admittance to the pediatric ward. Children, critically ill at admission, with significant comorbidity (including HIV), a history of anti-tuberculosis treatment (ATT) and/or contact with a TB case with known drug resistance, were excluded. According to the National TB Control Program, household siblings of the included TB cases were investigated for co-comittant disease. Following a study amendment to enable the present sub-study of biomarkers, asymptomatic siblings were eligible for the purpose of the present study after exclusion of TB.

### Diagnostic assessment

Medical history (including BCG vaccination status, history of TB and/or TB exposure), clinical (including current symptoms and physical examination), demographic, and anthropometric data were recorded. A TST was performed by a trained nurse (5 TU/0.1mL tuberculin; Span Diagnostics, Surat, India) and read after 48–72 hours; an induration ≥10 mm was defined as positive. Peripheral blood (3 ml) was drawn for the QuantiFERON®-TB Gold In-Tube (QFT) (Cellestis, Australia) which was performed according to the manufacturer’s instructions (not done in asymptomatic siblings). A chest X-ray, anterioposterior and lateral view (CXR), were recorded and interpreted by 3 independent radiologists. Agreement by 2 radiologists was required for a diagnosis of probable intra-thoracic TB (findings of hilar or mediastinal adenopathy, consolidation, cavity, miliary shadows, and/or pleural effusion; criteria similar to the diagnostic recommendation by the revised NTPs)^31^. From suspected TB cases (not asymptomatic siblings), gastric aspirates and induced sputa were collected on 2 consecutive days for direct fluorescent microscopy (Auramine) and culture (Mycobacterial Growth Indicator Tube, BD) and processed as described previously^32^. The prevalence of HIV infection was assessed anonymously as part of the main study (TB cases only) and found to be <1%.

### Study population and case definitions

Of the 403 children with intra-thoracic TB included in the main study, the last 100 were asked to participate in the present study, which was made possible after additional financing an protocol amendment. Eighty-eight consented to an additional blood draw (PAXgene Blood RNA Tubes; PreAnalytiX, Hilden, Germany) and were subsequently available for biomarker analysis: 40 smear/culture positive and 48 smear/culture negative for MTB.

Following the protocol amendment, 80 household siblings were asked to participate in the study, but as we experienced a general reluctance by parents to a blood draw, especially in asymptomatic children, consent was only achieved for 39 household siblings. They differed with regard to the TST response: 15 TST-positive, 24 TST-negative (Fig. 1). Thirty of 39 were siblings to the 88 TB cases analyzed herein, of whom three cases had more than one sibling (3 or 4). Study flowchart is given in Fig. 1.

As our aim was to identify diagnostic immune biomarkers across the pediatric TB disease spectrum, children with intra-thoracic TB established on the basis of culture-confirmed TB (diagnostic gold standard) were considered to have most advanced disease and thus representative for the upper end of the TB disease spectrum (n = 40); followed by children with typical TB–related findings on CXR, but culture negative TB disease (n = 48); followed by MTB infected (as judged by TST ≥ 10 mm) but asymptomatic siblings (n = 15); and finally MTB uninfected and asymptomatic siblings (n = 24) representing the lower end of the spectrum with possible successful clearance of MTB infection by innate immune mechanisms prior to induction of adaptive immunity.

### RNA extraction

Total RNA was extracted from the PAXgene blood collection tubes using the ‘PAXgene Blood RNA kit’ with RNase free DNase on-column digestion (PreAnalytiX, Hilden, Germany) according to the manufacturer’s instructions. The total RNA concentration and purity (A_260/280_nm ratio) were measured using a Nanodrop spectrophotometer (Thermoscientific, Wilmington, Delaware, U.S.A) and ranged between 0.2–13.7 μg (average 2.1 ± 0.64 μg) and supplemented by agarose gel electrophoresis.

### Dual colour Reverse Transcriptase-Multiplex Ligationdependent Probe Amplification (dcRT-MLPA)

As the blood sample available from children is limited, we used a novel high-throughput technique, which requires 130–150 ng of total RNA for a predefined panel of genes. The dcRT-MLPA protocol has been described in detail^15^, and therefore is briefly discussed here. A table of the 49 analyzed genes transcriptomes and their likely relation to TB pathogenesis are listed in Supplementary Table 1. Probes and primers were obtained from the Department of Infectious Diseases, Leiden Medical University, Leiden, The Netherlands. Samples with a concentration <50 ng/μl were concentrated at 45 ^o^C using a speed-vacuum concentrator (Eppendorf AG, Hamburg, Germany). A positive control (using synthetic oligonucleotides as hybridization templates) and a commercial Human Universal Reference RNA were included on each plate. All samples (n = 127) were run in duplicates. The amplified PCR products were diluted 1:10 with nuclease-free H_2_O and added to a mixture of Hi-Di-Formamide with 400HD ROX size standard. The PCR fragments were denatured at 95 ^o^C for 5 min, cooled on ice and analyzed on a 3730 capillary sequencer (Life Technologies, Carlsbad, California, USA). Data were analyzed using GeneMapper version 4.0 (Life Technologies, Carlsbad, California, USA) with adjustments of the default peak detection settings if required. The peak area of replicates were averaged, normalized against GAPDH and log2 transformed as described^17^. Of the 48 genes, 12 had expression levels below the threshold of detection 7.64 (peak area <200 arbitrary units), and therefore were omitted from further analysis.

### Multiplex bead array (bioplex)

Biomarkers at the translational level were analyzed in peripheral WB stimulated with MTB-specific antigens: Early Secretory Antigenic Target-6 (ESAT-6), Culture Filtrate Protein-10 (CFP10) and TB antigen 7.7 (QFT supernatants) when available (n = 80, TB cases only) and analyzed using the ‘Human cytokines 18-plex’ kit (Bio-Rad Laboratories Inc., Hercules, California, USA) according to the manufacturer’s instructions.

### Statistical analysis

Differences in clinical characteristics between the study groups were assessed by Pearson’s chi-square test or Fisher’s exact test, where appropriate.

Differences in single transcriptional (dcRT-MLPA) and translational biomarkers (bioplex) between the clinical groups were assessed by means of pair-wise comparisons while controlling for age (in months) using either linear mixed model analysis (also controlling for sibling-specific random effects), or linear regression model where appropriate (e.g., there were no siblings among TB cases and household controls). IBM SPSS version 21 was used (IBM, Bergen, Norway). Differences in single translational biomarkers (bioplex) between the clinical groups were evaluated by means of a parametric event-time model assuming a log-normal distribution and including adjustment for age and gender. This model allowed the large proportion of left- and right-censored biomarker levels to be included in the analysis but receiving less weight than fully observed levels. The statistical environment R was use for the analyses (R Core Team, 2014). Dot plots were created using GraphPad Prism 5 (GraphPad Software, Inc. La Jolla, California, USA).

Global differences in biomarker profiles (transcriptional and translational) were assessed pair-wise by two approaches: 1) a global test for providing a hierarchical clustering of genes based on average linkage and absolute correlation distance^19^, 2) Least absolute shrinkage and selection operator (lasso) regression with covariate adjustment for age^33^. The discriminatory power of the identified biomarker signatures was evaluated for sensitivity and specificity using area under curve (AUC) of the resulting receiver operating characteristic (ROC) curve. Both approaches were performed using R (R Core Team 2014).

### Ethics statement

Written informed consent was obtained from parents/guardians as well as written assent for participants >7 years. The study protocol was approved by the Institute Ethics Committee, All India Institute of Medical Sciences, New Delhi, and the Institutional Ethical Committee, Lady Hardinge Medical College, New Delhi, India. All experiments were performed in accordance with relevant guidelines and regulations. Details of the double-blind randomized control trial within which this biomarker sub-study is nested was registered at clinicaltrials.gov (NCT00801606).

## Additional Information

**How to cite this article**: Jenum, S. *et al.* Approaching a diagnostic point-of-care test for pediatric tuberculosis through evaluation of immune biomarkers across the clinical disease spectrum. *Sci. Rep.* **6**, 18520; doi: 10.1038/srep18520 (2016).

## Supplementary Material

Supplementary Information

## Figures and Tables

**Table 1:** Characteristics in children with TB disease.

	Culture + n = 40 (%)	Culture −n = 48 (%)
Demographics		
Age in months (mean)	111.5	104
Range	97–126	93–116
Gender (male)	15 (38)	23 (48)
Mycobacterial exposure		
Known BCG vaccination°	32 (80)	42 (88)
Known TB exposure¹	14 (35)	16 (33)
Tuberculin Skin Test		
Positive (≥10 mm)	39 (98)	46 (96)
Median (mm)	18	19
QuantiFERON Gold In-tube		
Positive (≥0.35 IU/mL)	30 (75)	36 (75)
Indeterminate	1 (2.5)	0
Median (IU/mL)	2.0	6.0
Symptoms		
Cough≥2 weeks^2^	34 (85)	22 (46)
Fever≥2 weeks^2^	36 (90)	35 (73)
Weight Loss	36 (90)	29 (60)
Findings		
Abnormal Chest X-ray	40 (100)	48 (100)
BMI-for-age percentile<5^3^	26 (65)	30 (63)

**Table 2:** Characteristics of asymptomatic household siblings with normal chest X-ray.

	TST + n = 15 (%)	TST–n = 24 (%)
Demographics		
Age in months (mean)	109	106
Range	71–146	86–125
Gender (male)	9 (60)	13 (54)
Mycobacterial exposure		
BCG vaccinated sibling with TB disease	13 (87)	19 (80)
Any TB exposure	15 (100)	24 (100)
Exposure to adult TB case	0	7 (29,100*)
Relationship with index case	–	
Primary caregiver		2 (8, 29*)
Relative in household		4 (17, 57*)
Not within household		1 (4, 14*)
Average exposure per day	–	
≥12 hours		6 (25, 86*)
4–7 hours		1 (4, 14*)
Average duration of exposure	–	
≥12 weeks		7 (29, 100*)
Findings		
BMI-for-age percentile <5^3^	5 (33)	4 (17)
missing data	4 (27)	7 (29)

°BCG - Bacillus Calmette Guerin. ¹Known TB exposure from any TB case. ^2^No improvement during a 7–10 day course of amoxicillin. ^3^Body Mass Index-for-age percentile range: Underweight < 5; Normal weight ≥ 5 and < 85; Overweight ≥ 85 and < 95; Obesity ≥ 95. *Percent of siblings with known exposure, n = 7.

**Table 3:**
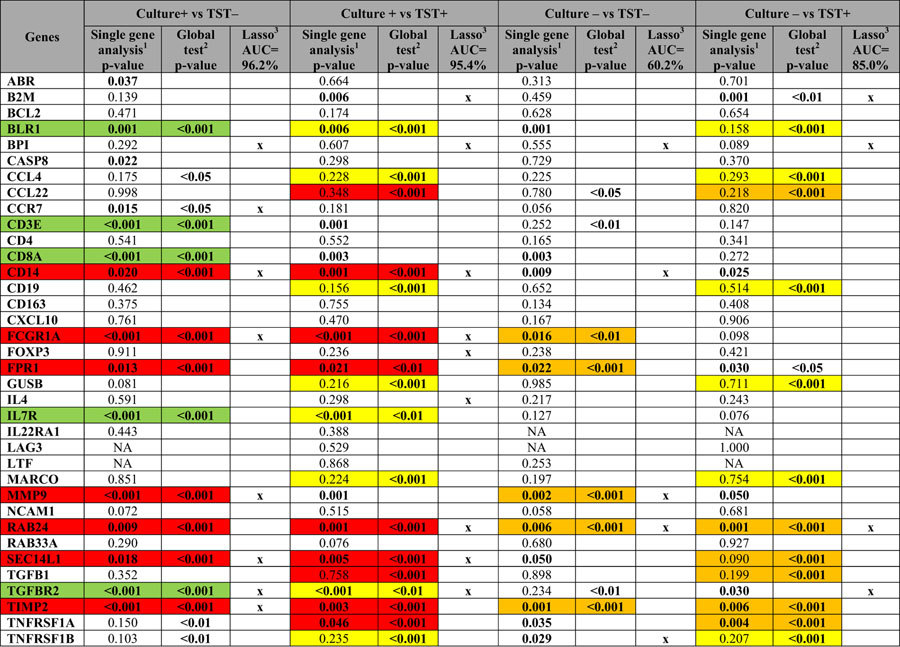
dcRT-MLPA based gene comparisons for the TB cases (culture + , culture–) and asymptomatic household siblings (TST + and TST–).

^1^Linear mixed model with adjustment for age and the random effect of siblings. p value ≤ 0.05 were considered to be significant. The significant p-value is highlighted. ^2^Global test without controlling for age and the random effect of siblings. Only biomarkers included in the hierarchical cluster significantly expressed between the clinical groups are illustrated. ^3^Lasso regression, only adjusted for age, was performed for all comparisons to select the set of biomarkers (biosignature) with the best discriminatory power between the study groups. Biomarkers retained and contributing to the area under the curve (AUC) shown at the top are indicated by x. 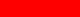 highest gene expression associated with culture-positive for MTB. 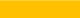 highest gene expression associated with culture-negative for MTB. 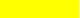 highest gene expression associated with asymptomatic TST + household siblings. 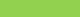 highest gene expression associated with asymptomatic TST**–** household siblings.

**Table 4:**
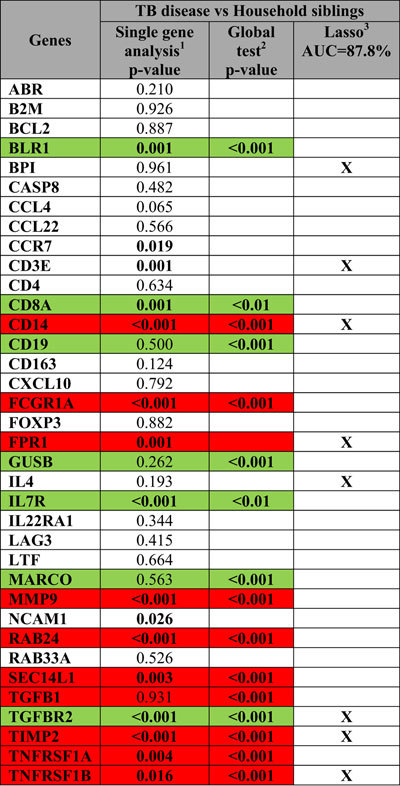
Comparisons of single gene expression between TB cases (regardless of culture result) and asymptomatic household siblings (regardless of TST result).

^1^Linear mixed model with adjustment for age and the random effect of siblings. p value < 0.05 were considered to be significant. The significant p-value is highlighted. ^2^Global test without controlling for age and the random effect of siblings. Only biomarkers included in the hierarchical cluster significantly expressed between the clinical groups are illustrated. ^3^Lasso regression, only adjusted for age, was performed for all comparisons to select the biomarker signature with the best discriminatory power between the study groups. Biomarkers predicted in model having the defined area under the curve (AUC) are shown and indicated by x. 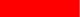 highest gene expression associated with TB disease (culture + and culture**–**). 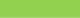 highest gene expression associated with household siblings (TST + and TST**–**).
