# Supplementary material for: Approaching a diagnostic point-of-care test for pediatric tuberculosis through evaluation of immune biomarkers across the clinical disease spectrum
Source: Sci Rep. 2016 Jan 4;6:18520. doi: 10.1038/srep18520 (PMC4698754; doi:10.1038/srep18520)
Supplement: Supplementary Information [file srep18520-s1.pdf]

Supplementary information

"Approaching a diagnostic point-of-care test for pediatric tuberculosis through evaluation of immune biomarkers across the clinical disease spectrum."

Running title: Approaching a POC-test for pediatric TB

Co-authors: Synne Jenum[ƚ1], Dhanasekaran S[ƚ2], Rakesh Lodha*[3], Aparna Mukherjee[3], Deepak Kumar Saini[3], Sarman Singh[4], Varinder Singh[5], Guruprasad Medigeshi[3], Marielle C Haks[6], Tom HM Ottenhoff[6], T Mark Doherty[7], SK Kabra[3], Christian Ritz[8] and Harleen MS Grewal*[2,2a].

Authors' Addresses: [1]*Department of Global Public Health and Primary Care, University of Bergen, and Department of Medical Microbiology, Vestre Viken Hospital Trust, Drammen, Norway.* [2]*Department of Clinical Science, Faculty of Medicine and Dentistry, University of Bergen, N-5021, Norway.* [2a]*Department of Microbiology, Haukeland university hospital, University of Bergen,N-5021, Norway.* [3]*Department of Pediatrics, All India Institute of Medical Sciences, New Delhi, India.* [4]*Division of Clinical Microbiology & Molecular Medicine, Department of Laboratory Medicine, All India Institute of Medical Sciences, New Delhi, India.* [5]*Department of Pediatrics, Kalawati Saran Children Hospital, New Delhi, India.* [6]*Department of Infectious Diseases Group, Immunology and Immunogenetics of Bacterial Infectious Disease, Leiden University Medical Center, The Netherland.* [7]*GlaxoSmithKline Pharma, Vaccines, Brøndby , Denmark.* [8]*Department of Nutrition, Exercise and Sports, University of Copenhagen, Denmark.*

[ƚ]**Equal contribution**

***CORRESPONDING AUTHORS**

Dr. Rakesh Lodha

Department of Pediatrics

AIIMS New Delhi

Phone: +91-11-26594610; Fax : +91-11-26588663, +91-11-26588641

Email- rlodha1661@gmail.com

Harleen M.S Grewal MD, PhD, DTMH

Professor and Senior Consultant,

Department of Clinical science infection, Faculty of Medicine and Dentistry

University of Bergen & Department of Microbiology

Haukeland University Hospital

Bergen 5021, Norway.

Phone: +47 55-97-4631; Mobile: +47 99450554; Fax: +47 55-97-4689

Email- Harleen.Grewal@.uib.no

Supplementary Table 1: Genes investigated in the dcRT-MLPA and their functions

Genes	Putative role	Reference
**BCL2**	B-cell lymphoma 2, down-regulates cell death (apoptosis). Increased *BCL2* expression in infected macrophages may create an immune privileged site for MTB infection and is implicated in poor prognosis for TB patients.	1,2
**BLR1**	Belongs to CXC chemokine receptor family (CXCR5), located in lymph node follicles. An increased expression of *BLR1* in TB patients might help in sustaining the expression of its ligand CXCL13, which in turn attracts B cells.	3
**BPI**	Bactericidal permeability-increasing protein, found in neutrophil granules and associated with host defence through microbiocidal activity.	4
**CASP8**	Cysteine-aspartic acid protease family, plays a central role in the execution-phase of cell apoptosis and increased expression is seen in TB patients	5,6
**CCL4**	Chemokine (c-c motif) ligand 4 or Macrophage inflammatory protein 1β (MIP-1β), acts as chemo-attractant for a variety of immune cells.	7
**CCL13**	Chemokine (c-c motif) ligand 13, contributes to control of MTB infection through homing of lymphocytes and dendritic cells.	8
**CCL19**	Chemokine (c-c motif) ligand 19, has a similar function to CCL13.	8
**CCL22**	Belongs to CC chemokine family, plays a role in the trafficking of activated T cells to inflammatory sites.	7
**CCR7**	CC chemokine receptor 7, mediates trafficking of dendritic cells and T cells from the lungs to the mediastinal lymph node during TB disease.	9,10
**CD3E**	T-cell glycoprotein CD3 epsilon, plays a role in intracellular signal-transduction pathways.	11
**CD4**	Cluster of differentiation 4, plays a critical role in adaptive immunity to TB.	12
**CD8A**	Cluster of differentiation 8A, identifies cytotoxic T- cells that interact with MHC class I targets and may contribute to control of MTB infection.	13
**CD14**	Cluster of differentiation 14, a component of the innate immune system and acts a co-receptor for recognizing MTB. Monocyte/macrophage marker.	14,15
**CD19**	Cluster of differentiation 19, a B cell marker and essential for B cell activation.	16
**CD163**	Cluster of differentiation 163, a hemoglobin scavenger receptor exclusively expressed in macrophages upon inflammation.	17
**CTLA4**	Cytotoxic T-lymphocyte antigen 4 plays a role in the down-regulation of T cell immune responses and involved in the maintenance of T cell homeostasis. *CTLA4* gene expression may be associated with severity of pulmonary TB.	18,19
**CXCL10**	CXC chemokine 10 or interferon gamma-induced protein 10 (IP-10), secreted by number of cell types in response to IFN-α/β and involved in stimulation of natural killer cells and T-cell migration in MTB infection.	20
**FASLG**	Fas ligand, a type II transmembrane protein, belonging to the TNF family involved in immune modulation and pathogenesis of TB. Modulators of Fas/FasL-mediated apoptosis may therefore be clinically useful.	21
**FCGR1A**	The Fc region of immunoglobulin gamma is involved in both innate and adaptive immune responses. It aids to control MTB infection and plays a central role in antibody-dependent cytotoxicity and the clearance of immune complexes.	22
**FOXP3**	Forkhead box P3, belongs to the transcription factor family and is involved in various cellular processes, acts as an important regulator for T-cell development.	23
**FPR1**	Formyl peptide receptor 1, a member of G-protein-coupled receptor family involved in the control of inflammation and neutrophil function.	24
**IFNγ**	Interferon gamma, secreted by several cell types such as NK cells, $CD4^+$ and $CD8^+$ T-cells, essential for control of MTB infection.	25

**IL2RA**	Interleukin 2 receptor gene, plays an important role in the activation and expansion of T-cells.	26
**IL4**	Interleukin 4, the prototypical Th2 cytokine; high levels of expression are associated with poor outcomes in TB.	27
**IL4d2**	IL-4 antagonist and splice variant of interleukin-4.	28
**IL7R**	Interleukin-7 receptor, plays a role in the development of immune cells and control of apoptosis.	29
**IL10**	Interleukin-10, an anti-inflammatory cytokine that may contribute to TB pathogenesis. IL-10 blocks phagosome maturation by a STAT3-dependent, p38-independent mechanism, which facilitates MTB survival and outgrowth	30
**IL22RA1**	Interleukin 22 receptor alpha 1belongs to class II cytokine receptor family and activates various signaling pathways. The expression of *IL22RA1* was found to be higher in late TB granulomas.	31,32
**LAG3**	Lymphocyte-activation gene 3, an important regulatory molecule involved in controlling the expansion and activation of T-cells.	33
**LTF**	Lacto-transferrin also called Lactoferrin (LF), a secreted mediator that connects innate and adaptive immune responses.	34
**MARCO**	Antigen-presenting cell scavenger receptor, involved in TLR activation that mediates phagocytosis of pathogens.	35
**MMP9**	Matrix metallo-peptidase 9, induced by MTB infection and has a role together with MCP-1in recruiting macrophages to the lungs during granuloma formation.	36
**NCAM1**	Neural cell adhesion molecule 1, mediates several intracellular signaling pathways and may be involved in TB pathogenesis.	37,38
**RAB13**	Ras related protein-13, a small GTPase family member, regulates assembly of functional tight junctions in epithelial cells.	39
**RAB24**	Ras related protein-24, a small GTPase family member, regulates intracellular protein trafficking between endoplasmic reticulum and cis-Golgi compartment.	39
**RAB33A**	Ras related protein-33A, a small GTPase family member; Dysregulation of GTPase plays a role in blocking of phagosome maturation, which is a major survival strategy for MTB.	40
**SEC14L1**	SEC14 cytosolic factor family plays a role in lipid metabolism and the intracellular transport system. SEC14L1 also appears to be a negative regulator of some innate immune functions.	41,42
**SPP1**	Secreted phosphoprotein 1, acts as a cytokine that up-regulates the expression of interferon-gamma and interleukin-12, contributes to resistance against mycobacteria by boosting reactive oxygen intermediate production in macrophages.	43,44
**TGFB1**	Transforming growth factor β1, an anti-inflammatory cytokine, performs many cellular functions and is involved in resolution of granulomatous lesions in TB.	45
**TGFBR2**	Transforming growth factor β receptor 2, involved in signal transduction and response to inhibit cell growth and division.	46
**TIMP2**	Tissue inhibitor of metallo-proteinases, involved in pathological changes, tissue remodeling and possibly, pathogenesis of pulmonary TB.	47
**TNF**	Tumor necrosis factor, a cytokine that plays multiple roles in the immunopathology of TB and is essential for controlling MTB infection.	48
**TNFRSF1A**	TNF receptor superfamily member1A, mediates apoptosis and functions as a regulator of inflammation.	49
**TNFRSF1B**	TNF receptor superfamily member 1B, mediates anti-apoptotic signals.	49
**TNFRSF18**	TNF receptor superfamily member 18, involved in T-cell activation, programmed cell death and pulmonary fibrosis.	47,49
**ABR**	Active BCR-Related gene contains a GTPase-activating protein domain (used as endogenous control).	
**B2M**	β2 microglobulin, a component of MHC class I molecules (used as endogenous control).	
**GAPDH**	Glyceraldehyde 3-phosphate dehydrogenase, involved in quite a few non-metabolic processes (used as endogenous control).	
**GUSB**	Glucuronidase β, regulates lysosomal storage function and co-regulated in response to stress (used as endogenous control).	50

References for Supplementary Table 1

1 Mogga, S. J., Mustafa, T., Sviland, L. & Nilsen, R. Increased Bcl-2 and reduced Bax expression in infected macrophages in slowly progressive primary murine Mycobacterium tuberculosis infection. *Scand J Immunol* **56**, 383-391 (2002).

2 Elliott, T. O. *et al.* Dysregulation of Apoptosis Is a Risk Factor for Tuberculosis Disease Progression. *The Journal of infectious diseases*, doi:10.1093/infdis/jiv238 (2015).

3 Mihret, A. *et al.* Combination of gene expression patterns in whole blood discriminate between tuberculosis infection states. *BMC infectious diseases* **14**, 257, doi:10.1186/1471-2334-14-257 (2014).

4 Schultz, H. & Weiss, J. P. The bactericidal/permeability-increasing protein (BPI) in infection and inflammatory disease. *Clinica chimica acta; international journal of clinical chemistry* **384**, 12-23, doi:10.1016/j.cca.2007.07.005 (2007).

5 Elmore, S. Apoptosis: a review of programmed cell death. *Toxicologic pathology* **35**, 495-516, doi:10.1080/01926230701320337 (2007).

6 Sloot, R. *et al.* Biomarkers Can Identify Pulmonary Tuberculosis in HIV-infected Drug Users Months Prior to Clinical Diagnosis. *EBioMedicine* **2**, 172-179, doi:10.1016/j.ebiom.2014.12.001 (2015).

7 Le, Y., Zhou, Y., Iribarren, P. & Wang, J. Chemokines and chemokine receptors: their manifold roles in homeostasis and disease. *Cellular & molecular immunology* **1**, 95-104 (2004).

8 Khader, S. A. *et al.* In a murine tuberculosis model, the absence of homeostatic chemokines delays granuloma formation and protective immunity. *Journal of immunology* **183**, 8004-8014, doi:10.4049/jimmunol.0901937 (2009).

9 Olmos, S., Stukes, S. & Ernst, J. D. Ectopic activation of Mycobacterium tuberculosis-specific CD4+ T cells in lungs of CCR7-/- mice. *Journal of immunology* **184**, 895-901, doi:10.4049/jimmunol.0901230 (2010).

10 Haynes, N. M. *et al.* Role of CXCR5 and CCR7 in follicular Th cell positioning and appearance of a programmed cell death gene-1high germinal center-associated subpopulation. *Journal of immunology* **179**, 5099-5108 (2007).

11 Banner, B., Spicer, Z. & Alroy, J. Expression of CD3 epsilon subunit in gastric parietal cells: a possible role in signal transduction? *Pathology, research and practice* **199**, 137-143 (2003).

12 Joosten, S. A. *et al.* Identification of biomarkers for tuberculosis disease using a novel dual-color RT-MLPA assay. *Genes and immunity* **13**, 71-82, doi:10.1038/gene.2011.64 (2012).

13 Lin, P. L. & Flynn, J. L. CD8 T cells and Mycobacterium tuberculosis infection. *Semin Immunopathol* **37**, 239-249, doi:10.1007/s00281-015-0490-8 (2015).

14 Wang, C. *et al.* Serum complement C4b, fibronectin, and prolidase are associated with the pathological changes of pulmonary tuberculosis. *BMC infectious diseases* **14**, 52, doi:10.1186/1471-2334-14-52 (2014).

15 Ayaslioglu, E. *et al.* The role of CD14 gene promoter polymorphism in tuberculosis susceptibility. *J Microbiol Immunol Infect* **46**, 158-163, doi:10.1016/j.jmii.2012.05.008 (2013).

16 Depoil, D. *et al.* CD19 is essential for B cell activation by promoting B cell receptor-antigen microcluster formation in response to membrane-bound ligand. *Nature immunology* **9**, 63-72, doi:10.1038/ni1547 (2008).

17 Moestrup, S. K. & Moller, H. J. CD163: a regulated hemoglobin scavenger receptor with a role in the anti-inflammatory response. *Annals of medicine* **36**, 347-354 (2004).

18 McCoy, K. D. & Le Gros, G. The role of CTLA-4 in the regulation of T cell immune responses. *Immunology and cell biology* **77**, 1-10, doi:10.1046/j.1440-1711.1999.00795.x (1999).

19 Wang, C. *et al.* Association of CTLA4 gene polymorphisms with susceptibility and pathology correlation to pulmonary tuberculosis in Southern Han Chinese. *Int J Biol Sci* **8**, 945-952, doi:10.7150/ijbs.4390 (2012).

20 Lande, R. *et al.* IFN-alpha beta released by Mycobacterium tuberculosis-infected human dendritic cells induces the expression of CXCL10: selective recruitment of NK and activated T cells. *Journal of immunology* **170**, 1174-1182 (2003).

21 Mustafa, T., Mogga, S. J., Mfinanga, S. G., Morkve, O. & Sviland, L. Significance of Fas and Fas ligand in tuberculous lymphadenitis. *Immunology* **114**, 255-262, doi:10.1111/j.1365-2567.2004.02080.x (2005).

22 Sutherland, J. S. *et al.* Differential gene expression of activating Fcgamma receptor classifies active tuberculosis regardless of human immunodeficiency virus status or ethnicity. *Clin Microbiol Infect* **20**, O230-238, doi:10.1111/1469-0691.12383 (2014).

23 Larson, R. P., Shafiani, S. & Urdahl, K. B. Foxp3(+) regulatory T cells in tuberculosis. *Adv Exp Med Biol* **783**, 165-180, doi:10.1007/978-1-4614-6111-1_9 (2013).

24 Dorward, D. A. *et al.* The role of formylated peptides and formyl peptide receptor 1 in governing neutrophil function during acute inflammation. *Am J Pathol* **185**, 1172-1184, doi:10.1016/j.ajpath.2015.01.020 (2015).

25 Fenton, M. J. *et al.* Induction of gamma interferon production in human alveolar macrophages by Mycobacterium tuberculosis. *Infection and immunity* **65**, 5149-5156 (1997).

26 Milani, P. *et al.* Mechanics of the IL2RA gene activation revealed by modeling and atomic force microscopy. *PloS one* **6**, e18811, doi:10.1371/journal.pone.0018811 (2011).

27 Ashenafi, S. *et al.* Progression of clinical tuberculosis is associated with a Th2 immune response signature in combination with elevated levels of SOCS3. *Clin Immunol* **151**, 84-99, doi:10.1016/j.clim.2014.01.010 (2014).

28 Wassie, L. *et al.* Ex vivo cytokine mRNA levels correlate with changing clinical status of ethiopian TB patients and their contacts over time. *PloS one* **3**, e1522, doi:10.1371/journal.pone.0001522 (2008).

29 Akashi, K., Kondo, M. & Weissman, I. L. Role of interleukin-7 in T-cell development from hematopoietic stem cells. *Immunological reviews* **165**, 13-28 (1998).

30 O'Leary, S., O'Sullivan, M. P. & Keane, J. IL-10 blocks phagosome maturation in mycobacterium tuberculosis-infected human macrophages. *Am J Respir Cell Mol Biol* **45**, 172-180, doi:10.1165/rcmb.2010-0319OC (2011).

31 Mehra, S. *et al.* Transcriptional reprogramming in nonhuman primate (rhesus macaque) tuberculosis granulomas. *PloS one* **5**, e12266, doi:10.1371/journal.pone.0012266 (2010).

32 Lim, C. & Savan, R. The role of the IL-22/IL-22R1 axis in cancer. *Cytokine Growth Factor Rev* **25**, 257-271, doi:10.1016/j.cytogfr.2014.04.005 (2014).

33 Sierro, S., Romero, P. & Speiser, D. E. The CD4-like molecule LAG-3, biology and therapeutic applications. *Expert Opin Ther Targets* **15**, 91-101, doi:10.1517/14712598.2011.540563 (2011).

34 Siqueiros-Cendon, T. *et al.* Immunomodulatory effects of lactoferrin. *Acta pharmacologica Sinica* **35**, 557-566, doi:10.1038/aps.2013.200 (2014).

35 Komine, H., Kuhn, L., Matsushita, N., Mule, J. J. & Pilon-Thomas, S. Examination of MARCO activity on dendritic cell phenotype and function using a gene knockout mouse. *PloS one* **8**, e67795, doi:10.1371/journal.pone.0067795 (2013).

36 Taylor, J. L. *et al.* Role for matrix metalloproteinase 9 in granuloma formation during pulmonary Mycobacterium tuberculosis infection. *Infection and immunity* **74**, 6135-6144, doi:10.1128/IAI.02048-05 (2006).

37 Hortsch, M. & Umemori, H. *The sticky synapse: cell adhesion molecules and their role in synapse formation and maintenance*. (Springer, 2009).

38 Rivera-Marrero, C. A., Stewart, J., Shafer, W. M. & Roman, J. The down-regulation of cathepsin G in THP-1 monocytes after infection with Mycobacterium tuberculosis is associated with increased intracellular survival of bacilli. *Infection and immunity* **72**, 5712-5721, doi:10.1128/IAI.72.10.5712-5721.2004 (2004).

39 Jacobsen, M. *et al.* Ras-associated small GTPase 33A, a novel T cell factor, is down-regulated in patients with tuberculosis. *The Journal of infectious diseases* **192**, 1211-1218, doi:10.1086/444428 (2005).

40 Doherty, M., Wallis, R. S., Zumla, A. & group, W. H.-T. D. R. E. C. j. e. c. Biomarkers for tuberculosis disease status and diagnosis. *Current opinion in pulmonary medicine* **15**, 181-187 (2009).

41 Mousley, C. J., Tyeryar, K. R., Vincent-Pope, P. & Bankaitis, V. A. The Sec14-superfamily and the regulatory interface between phospholipid metabolism and membrane trafficking. *Biochim Biophys Acta* **1771**, 727-736, doi:10.1016/j.bbalip.2007.04.002 (2007).

42 Li, M. T. *et al.* Negative regulation of RIG-I-mediated innate antiviral signaling by SEC14L1. *J Virol* **87**, 10037-10046, doi:10.1128/JVI.01073-13 (2013).

43 Renkl, A. C. *et al.* Osteopontin functionally activates dendritic cells and induces their differentiation toward a Th1-polarizing phenotype. *Blood* **106**, 946-955, doi:10.1182/blood-2004-08-3228 (2005).

44 Khajoee, V. *et al.* Novel roles of osteopontin and CXC chemokine ligand 7 in the defence against mycobacterial infection. *Clin Exp Immunol* **143**, 260-268, doi:10.1111/j.1365-2249.2005.02985.x (2006).

45 Toossi, Z., Gogate, P., Shiratsuchi, H., Young, T. & Ellner, J. J. Enhanced production of TGF-beta by blood monocytes from patients with active tuberculosis and presence of TGF-beta in tuberculous granulomatous lung lesions. *Journal of immunology* **154**, 465-473 (1995).

46 Bellam, N. & Pasche, B. Tgf-beta signaling alterations and colon cancer. *Cancer treatment and research* **155**, 85-103, doi:10.1007/978-1-4419-6033-7_5 (2010).

47 Zhang, Q., Guo, Y., Dong, R., Dai, R. & Zhou, M. Suppressor of cytokine signaling 1-modulated metalloproteinases and tissue inhibitor of metalloproteinase in pulmonary fibrosis. *Mol Med Rep* **12**, 3855-3861, doi:10.3892/mmr.2015.3810 (2015).

48 Lin, P. L., Plessner, H. L., Voitenok, N. N. & Flynn, J. L. Tumor necrosis factor and tuberculosis. *J Investig Dermatol Symp Proc* **12**, 22-25, doi:10.1038/sj.jidsymp.5650027 (2007).

49 Croft, M. The role of TNF superfamily members in T-cell function and diseases. *Nature reviews. Immunology* **9**, 271-285, doi:10.1038/nri2526 (2009).

50 Dhanasekaran, S. *et al.* Identification of biomarkers for Mycobacterium tuberculosis infection and disease in BCG-vaccinated young children in Southern India. *Genes and immunity* **14**, 356-364, doi:10.1038/gene.2013.26 (2013).

Supplementary Table 2 dcRT-MLPA based gene comparisons between culture+ versus culture– and TST+ versus TST–.

Genes	Culture+ vs Culture–		
	Single gene analysis[1] p-value	Global test[2] p-value	Lasso Model[3] AUC=58.2%
ABR	0.560		
B2M	0.214		
BCL2	0.558		
**BLR1**	**0.002(**)**	**<0.001**	
BPI	0.305		x
CASP8	0.462		
CCL4	0.084		
CCL22	0.956		
CCR7	0.591		
CD3E	**0.050(*)**		x
CD4	0.513		
**CD8A**	**0.008(**)**	**<0.001**	
CD14	0.150		
CD19	0.542		
CD163	0.986		
CXCL10	0.471		
**FCGR1A**	**0.001(**)**	**<0.001**	x
FOXP3	0.879		
FPR1	0.152		
GUSB	0.232		
IL4	0.324		
**IL7R**	**0.003(**)**	**<0.01**	
IL22RA1	0.656		
LAG3	0.831		
LTF	0.227		
MARCO	**0.042(*)**	**<0.05**	
MMP9	0.069		
NCAM1	0.335		
RAB24	**0.013(*)**	**<0.01**	
RAB33A	0.321		
SEC14L1	0.318		
TGFB1	0.958		
**TGFBR2**	**0.001(**)**	**<0.001**	x
TIMP2	0.058	**<0.05**	
TNFRSF1A	0.475		
TNFRSF1B	0.498		

[1]Logistic regression analysis controlled for age. p-value ≤ 0.05 (*), <0.01 (**), <0.001 (***) were considered to be significant. The significant p-value is highlighted.

[2]Global test without controlling for age and the random effect of siblings. Only biomarkers included in the hierarchical cluster significantly expressed between the clinical groups are illustrated.

[3]Lasso regression model, controlled for age, was performed for all comparisons to select the set of biomarkers (biosignature) with the best discriminatory power between the study groups. Biomarkers predicted in the models having the defined area under the curve (AUC) are shown and indicated by x.

Red: highest gene expression associated with culture-positive for MTB

Orange: highest gene expression associated with culture-negative for MTB

Supplementary Figure Legends:

Supplementary Figure 1: A) illustrates the Global test comparisons based on dcRT-MLPA data. The binary outcomes for culture+ vs. TST+ are shown in the bar graph. B) illustrates the results from Lasso analysis of dcRT-MLPA data. The ability of biomarker signatures to predict clinical outcomes were identified following adjustment for age (months) in lasso regression analysis. The predicted probability of the identified biomarker signatures to discriminate between culture+ vs. TST+ is shown by: receiver operator characteristic curves (ROCs), area under the curve (AUC) and box-and-whisker plots (5–95 percentiles).

Supplementary Figure 2: A) illustrates the Global test comparisons based on dcRT-MLPA data. The binary outcomes for culture– vs. TST+ are shown in the bar graph. B) illustrates the results from Lasso analysis of dcRT-MLPA data. The ability of biomarker signatures to predict clinical outcomes were identified following adjustment for age (months) in lasso regression analysis. The predicted probability of the identified biomarker signatures to discriminate between culture– vs. TST+ is shown by: receiver operator characteristic curves (ROCs), area under the curve (AUC) and box-and-whisker plots (5–95 percentiles).

Supplementary Figure 3: Dot-plot graph depicting genes that are differentially expressed between the two clinical groups: TB disease vs. household siblings. Supplementary Figure 3A and 3B illustrate the median with inter quartile range relative gene expression (log 2 transformed) of genes from peripheral blood. p-value ≤ 0.05 (*), <0.01 (**), <0.001 (***) were considered to be significant.

Supplementary Figure 4: Illustrates the Global test comparisons based on dcRT-MLPA data. The binary outcomes for culture+ vs. culture– are shown in the bar graph.

1A

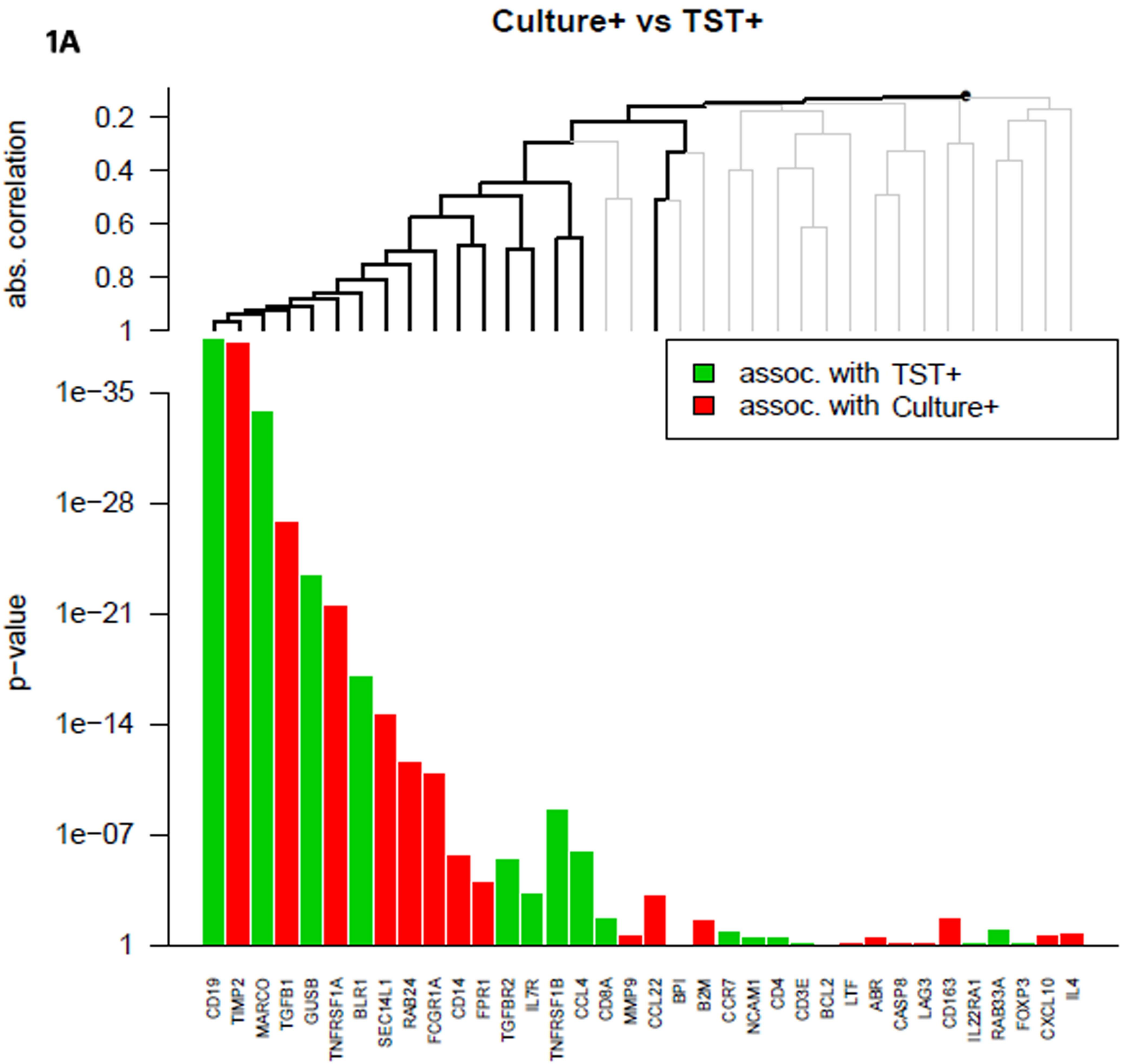

1B

(Intercept)	24.626540
Age	0.001105
BPI	-0.172685
FCGR1A	-0.475634
CD14	-0.176568
B2M	-1.341649
RAB24	-0.226419
SEC14L1	-0.062062
IL4	-0.320334
FOXP3	0.065269
TGFBR2	2.349021

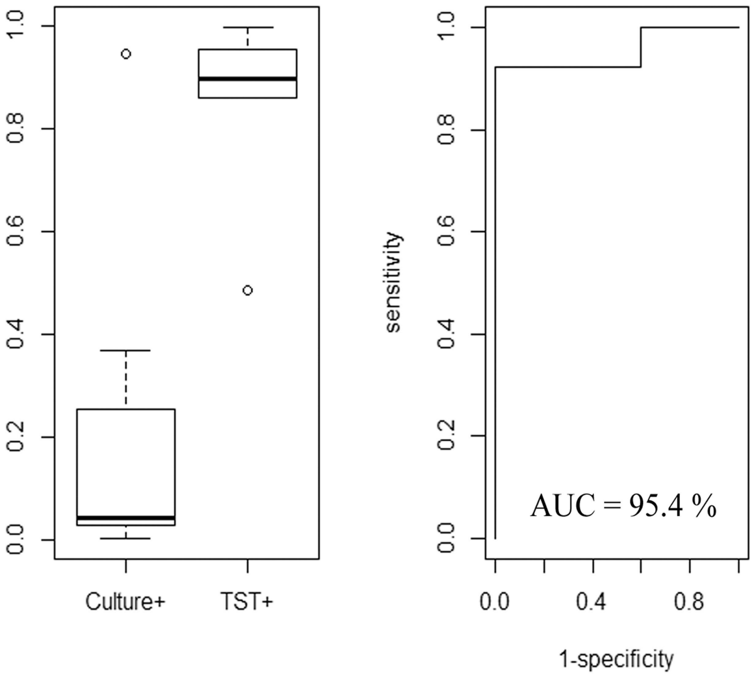

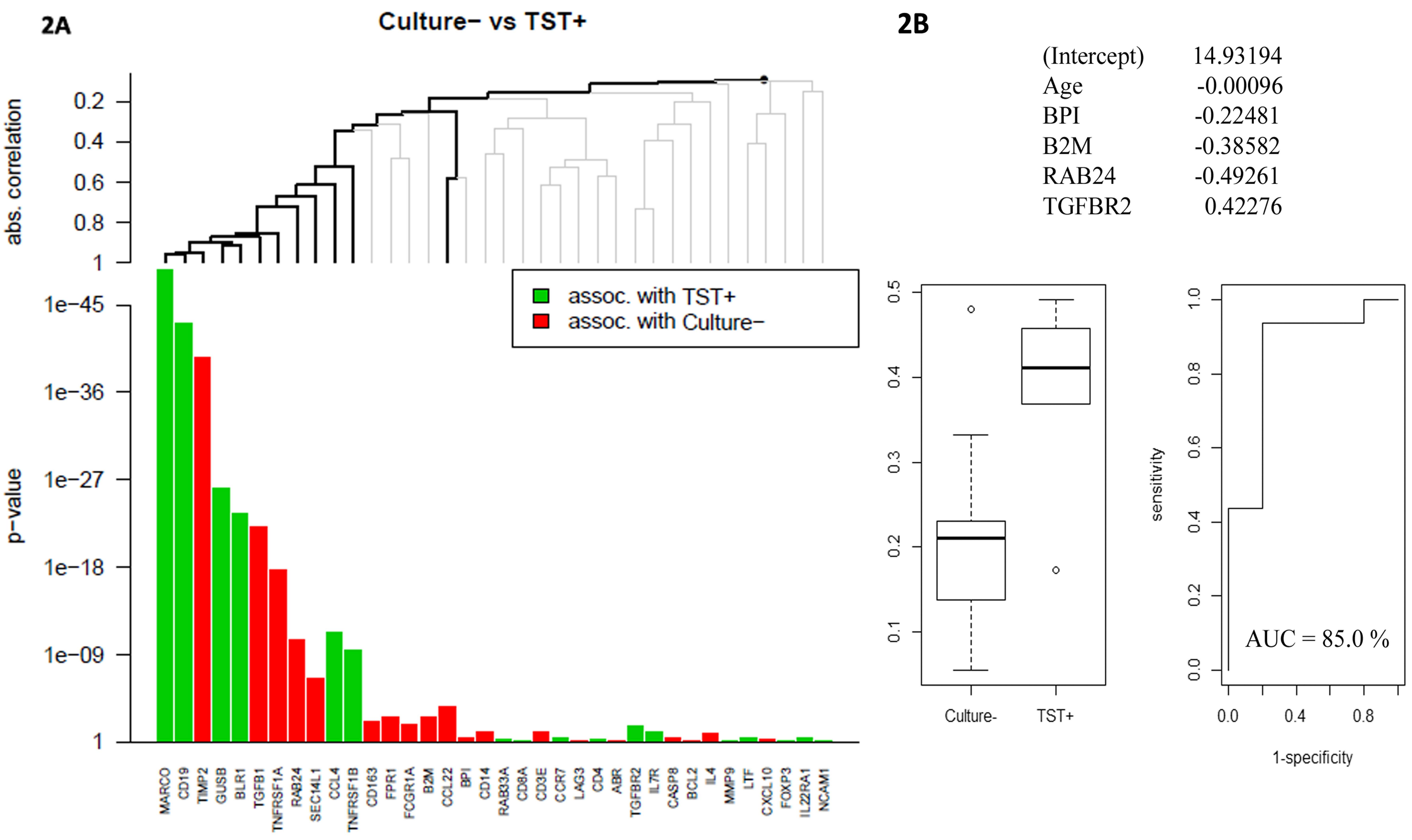

2A
Culture− vs TST+
abs. correlation
0.2
0.4
0.6
0.8
1
p−value
1e−45
1e−36
1e−27
1e−18
1e−09
1
assoc. with TST+
assoc. with Culture−
MARCO
CD19
TIMP2
GUSB
BLR1
TGFB1
TNFRSF1A
RAB24
SEC14L1
CCL4
TNFRSF1B
CD163
FPR1
FCGR1A
B2M
CCL22
BPI
CD14
RAB33A
CD8A
CD3E
CCR7
LAG3
CD4
ABR
TGFBR2
IL7R
CASP8
BCL2
IL4
MMP9
LTF
CXCL10
FOXP3
IL22RA1
NCAM1
2B
(Intercept) 14.93194
Age -0.00096
BPI -0.22481
B2M -0.38582
RAB24 -0.49261
TGFBR2 0.42276
0.1
0.2
0.3
0.4
0.5
Culture-
TST+
sensitivity
0.0
0.2
0.4
0.6
0.8
1.0
AUC = 85.0 %
1-specificity

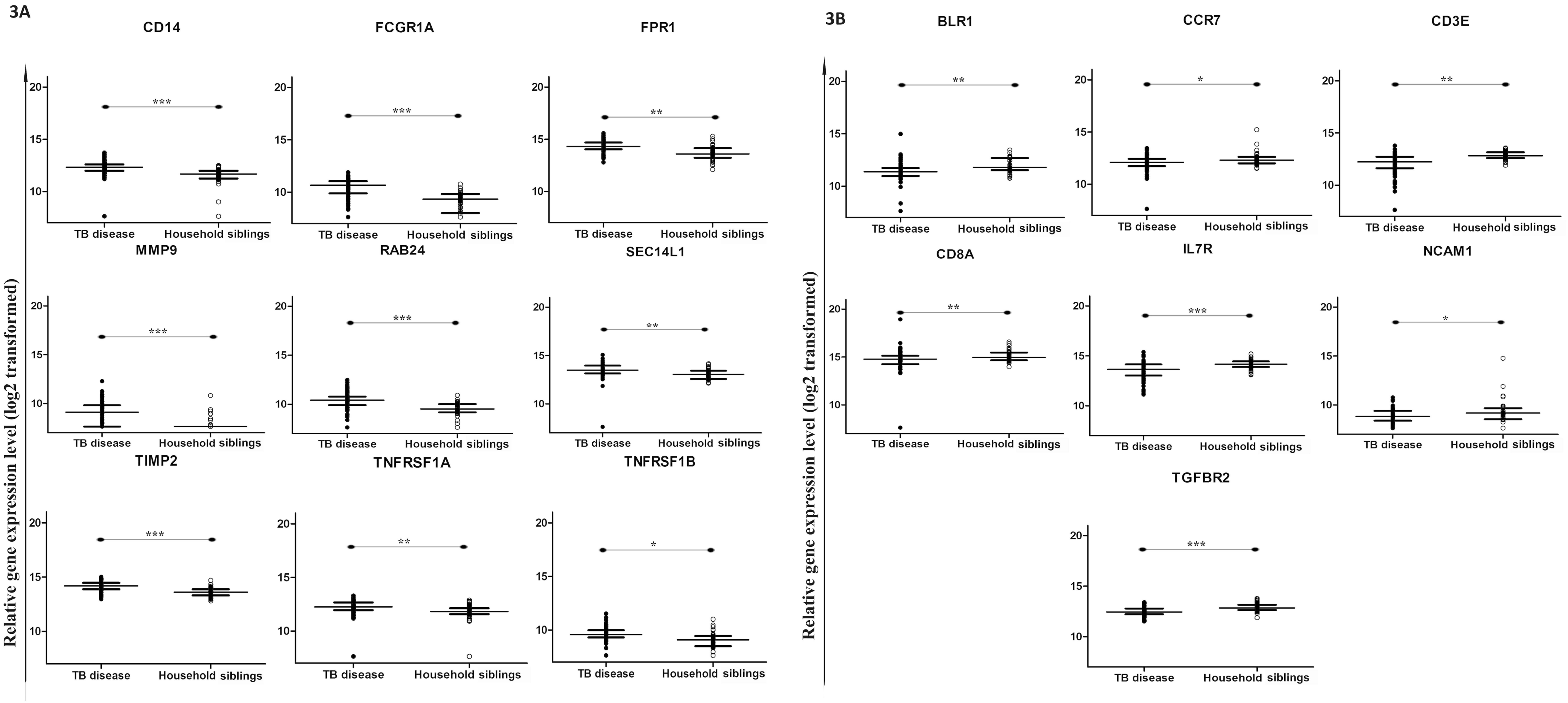
3A
CD14
FCGR1A
FPR1
MMP9
RAB24
SEC14L1
TIMP2
TNFRSF1A
TNFRSF1B
3B
BLR1
CCR7
CD3E
CD8A
IL7R
NCAM1
TGFBR2
Relative gene expression level (log2 transformed)
TB disease
Household siblings

4

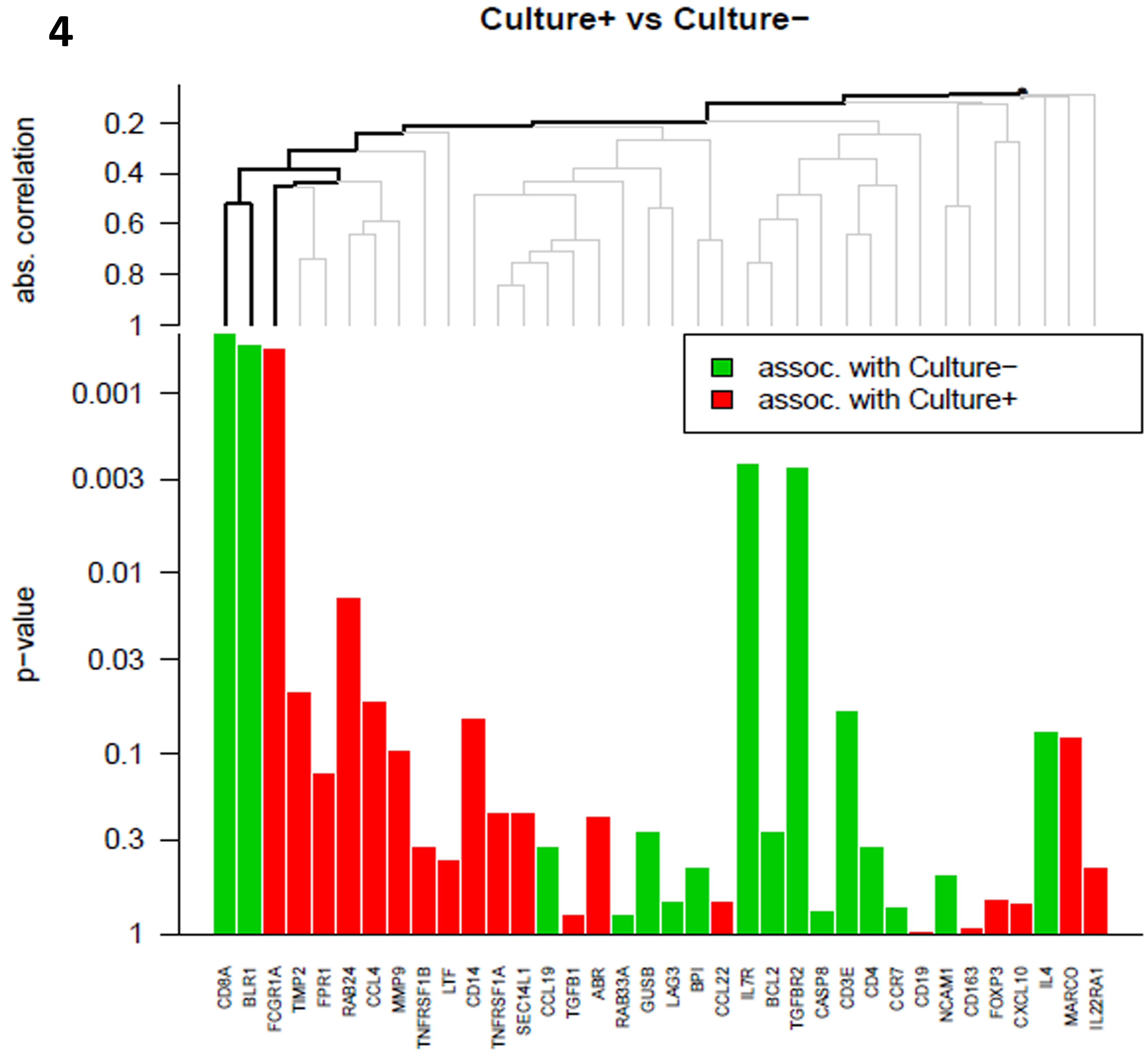
Culture+ vs Culture−
abs. correlation
0.2
0.4
0.6
0.8
1
p−value
0.001
0.003
0.01
0.03
0.1
0.3
1
assoc. with Culture−
assoc. with Culture+
CD8A
BLR1
FCGR1A
TIMP2
FPR1
RAB24
CCL4
MMP9
TNFRSF1B
LTF
CD14
TNFRSF1A
SEC14L1
CCL19
TGFB1
ABR
RAB33A
GUSB
LAG3
BPI
CCL22
IL7R
BCL2
TGFBR2
CASP8
CD3E
CD4
CCR7
CD19
NCAM1
CD163
FOXP3
CXCL10
IL4
MARCO
IL22RA1
